# An inexpensive and rapid diagnostic method of Koi Herpesvirus (KHV) infection by loop-mediated isothermal amplification

**DOI:** 10.1186/1743-422X-2-83

**Published:** 2005-10-17

**Authors:** Hatem Soliman, Mansour El-Matbouli

**Affiliations:** 1 Institute of Zoology, Fish Biology and Fish Diseases, Faculty of Veterinary Medicine, University of Munich, Germany

## Abstract

**Background:**

Koi Herpesvirus (KHV) affects both juvenile and adult common carp and koi, and is especially lethal to fry. The high mortalities caused by the disease have had a negative impact on the international koi trade. Different diagnostic techniques have been used to detect KHV, including: isolation of the virus in cell culture, electron microscopy, several PCR tests, ELISA and in situ hybridisation. All of these methods are time consuming, laborious and require specialised equipment.

**Results:**

A rapid field diagnosis of KHV in common and koi carp was developed using loop-mediated isothermal amplification (LAMP). The LAMP reaction rapidly amplified nucleic acid with high specificity and efficiency under isothermal conditions using a simple water bath. Two methods of extracting DNA from host tissue were compared: extraction by boiling and by using a commercial extraction kit. A set of six primers – two inner primers, two outer primers and two loop primers – was designed from a KHV amplicon. The reaction conditions were optimised for detection of KHV in 60 min at 65°C using *Bst *(*Bacillus stearothermophilus*) DNA polymerase. When visualised by gel electrophoresis, the products of the KHV LAMP assay appeared as a ladder pattern, with many bands of different sizes from 50 base-pairs (bp) up to the loading well. The KHV LAMP product could also be simply detected visually by adding SYBR Green I to the reaction tube and observing a colour change from orange to green. All samples positive for KHV by visual detection were confirmed positive by gel electrophoresis. The KHV LAMP had the same sensitivity as a standard PCR assay for the detection of KHV.

**Conclusion:**

This paper describes an accelerated LAMP assay for diagnosis of KHV. The entire procedure took only 90 minutes to produce a result: 15 minutes for DNA extraction; 60 min for the LAMP reaction; 2 min for visual detection using SYBR Green I. The test can be used under field conditions because the only equipment it requires is a water bath.

## Background

Koi Herpesvirus (KHV) is a highly contagious viral disease which causes significant morbidity and mortality in common carp (*Cyprinus carpio*) and its ornamental domesticated form, koi carp [1]. Although the virus is currently regarded as a DNA-virus belonging to family Herpesviridae [1], some reports have disputed this classification and have renamed the virus as Carp Nephritis and Gill Necrosis Virus, CNGV [2]. More recently, reports based on morphology and genetics have demonstrated strong evidence that KHV is indeed a herpesvirus [3].

The international trade in live fish is arguably the most effective dispersal pathway of fish diseases through incidental movement of pathogenic organisms [4].With respect to koi, exhibitions and national and international trading have facilitated the rapid global spread of KHV. The disease struck koi population in the USA and Israel in 1998 and spread rapidly [5]; it has been reported in Germany [6], Korea [7,8], Indonesia [9], Japan [10], South Africa, and Thailand (unpublished data).

Clinical signs of KHV are often non-specific and mortality may occur rapidly. Discoloration and severe necrosis of the gills is the most consistent sign of infection, with disorientation and erratically swimming prior to death, which can occur within 24–48 hours after the onset of clinical signs [11,12]. KHV has caused considerable economic losses in both the koi and carp culture industries: to fish breeders, retailers and hobbyists impacted by the cumulative mortalities associated with outbreaks [4,2]. There is a clear need for a reliable, rapid diagnostic procedure for the detection of KHV infection.

Rapid virological diagnosis through isolation of the virus has proven difficult and time consuming. A far more efficient approach is nucleic acid amplification; one of the most valuable tools in virtually all life science fields [13]. One of the most widely used techniques is the polymerase chain reaction (PCR) which uses heat denaturation of double-stranded DNA products to promote the next round of DNA synthesis [14,15]. A widely used PCR assay for KHV was developed [16], and a second PCR assay for KHV has been described [12]. A real-time TaqMan PCR assay for KHV has also been developed to detect and quantify KHV DNA in infected tissues [17]. While these PCR techniques have significantly increased our ability to detect KHV infection in koi and common carp, their requirement for a high precision thermacycler has prevented their widespread use in private clinics, for example, as a routine diagnostic tool.

A novel nucleic acid amplification method, loop-mediated isothermal amplification (LAMP), has been developed that does not require a theramcycler. LAMP relies instead on autocycling strand displacement DNA synthesis by a *Bst *DNA polymerase, to amplify DNA with high specificity, efficiency, and speed under isothermal conditions [13,18,19]. LAMP requires two specially designed inner and two outer primers to improve specificity [20,21]; if two additional 'loop' primers are added, the reaction time can be halved [20]. The amplification products are stem-loop DNA structures with several inverted repeats of the target, and cauliflower-like structures comprising multiple loops [22]. In the present study, we used a LAMP technique for diagnosis of KHV, and evaluated its sensitivity, specificity, and applicability.

## Results

### Optimisation of the KHV LAMP reaction

**Figure 2:**
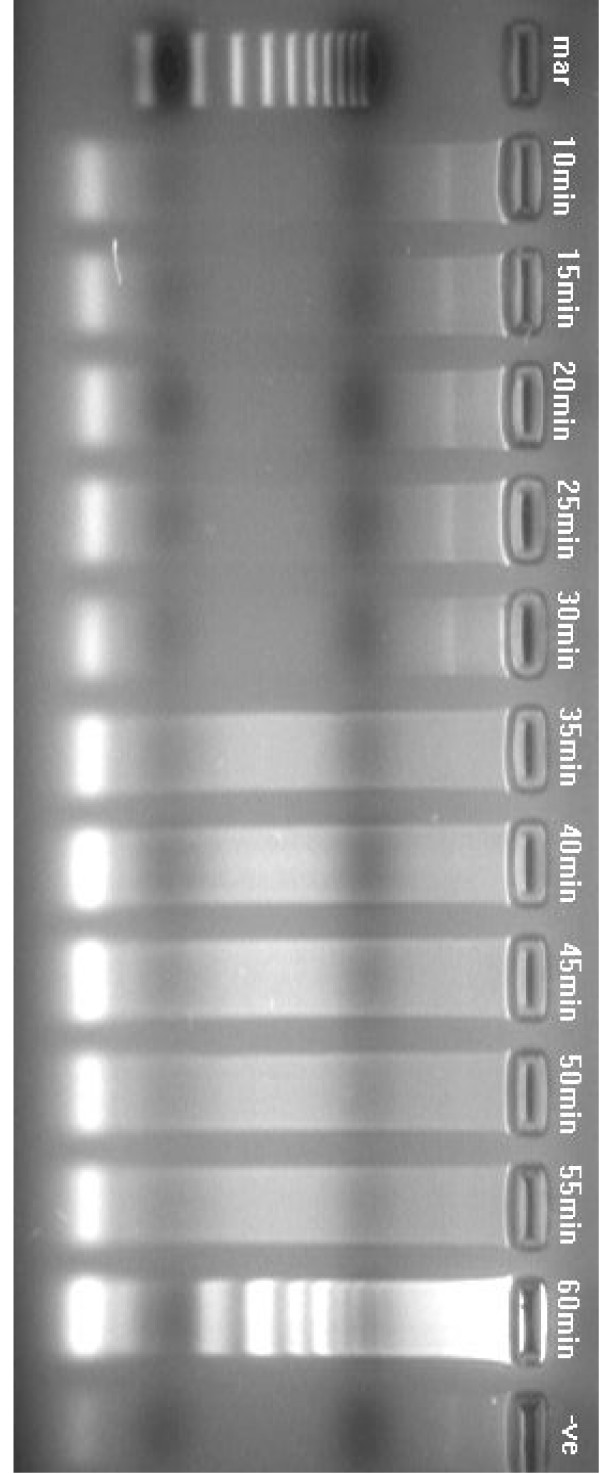
Agarose gel showing the effect of time on amplification of KHV DNA by LAMP assay, using four primers (FIP, BIP, F3, B3), carried out at 65°C for durations of 10–60 min. Lane mar = 100 bp DNA molecular weight standard, lane -ve = negative control. The LAMP assay detected KHV after 60 min.

**Figure 3:**
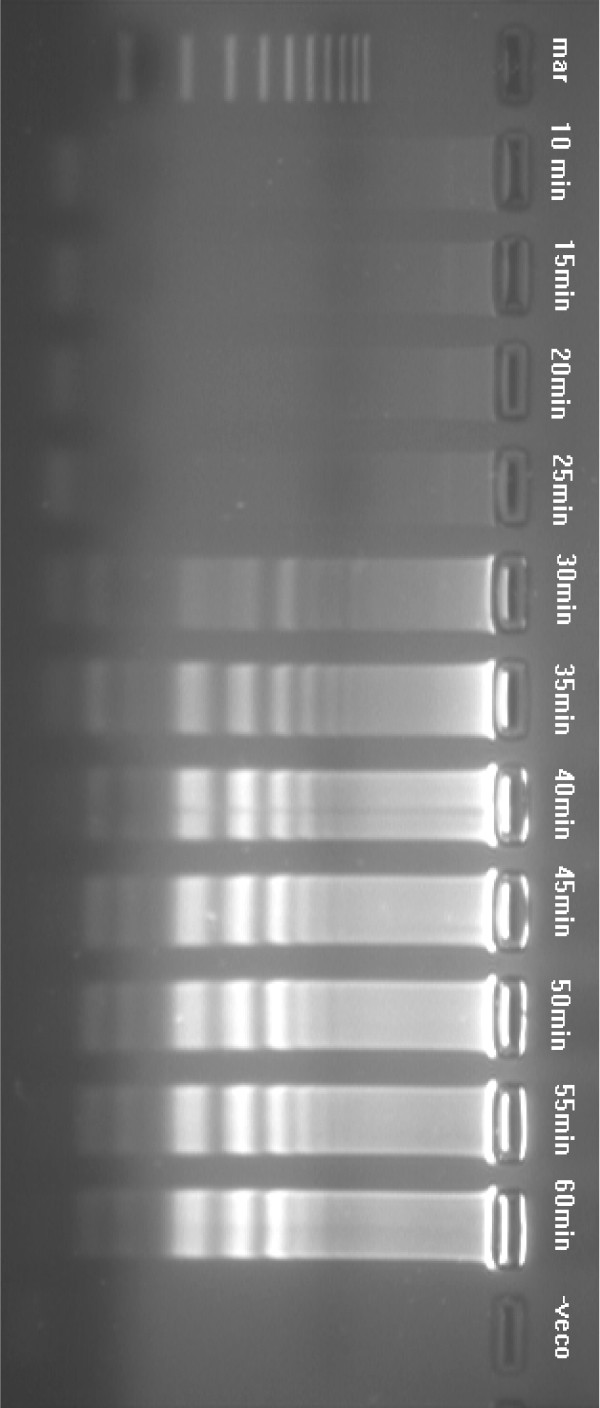
Agarose gel showing the effect of time on amplification of KHV DNA by LAMP assay using six primers (FIP, BIP, F3, B3, loopF, loopB), carried out at 65°C for durations of 10–60 min. Lane mar = 100 bp DNA molecular weight standard, lane -veco = negative control. The LAMP assay detected KHV as early as 30 min.

**Figure 4:**
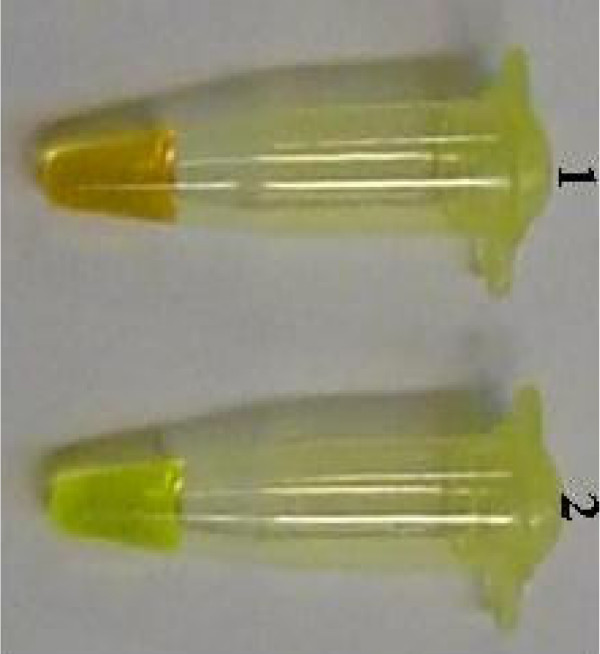
Visual detection of KHV LAMP products using SYBR Green I stain. 1: negative LAMP reaction remained orange. 2: positive LAMP reaction turned green.

**Figure 5:**
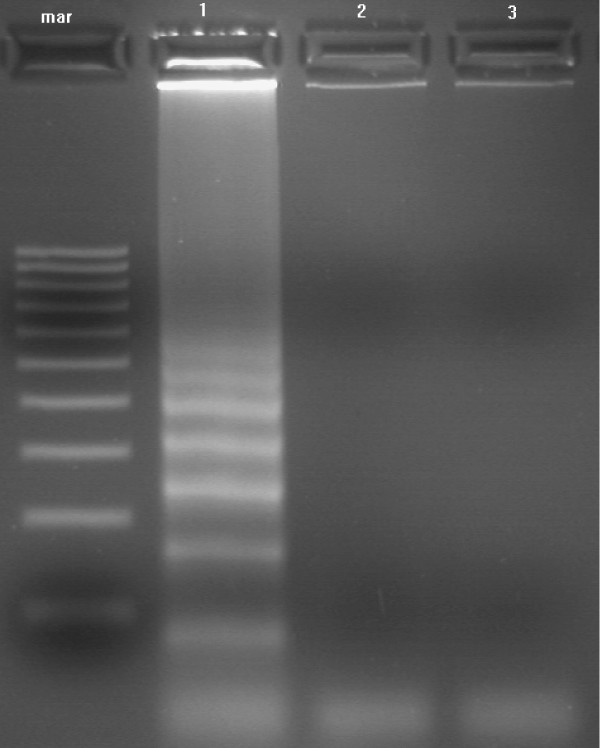
Agarose gel showing LAMP products of KHV DNA extracted by boiling. The reaction was carried out at 65°C using the 6 primer set. Lanes: mar = 100 bp molecular weight marker; 1 = KHV DNA extracted by boiling; 2 = negative fish tissue; 3 = negative control.

The LAMP reaction was performed using purified KHV genomic DNA as a template to determine the optimal primer combination and duration of reaction. The amplicon was formed using either 4 or 6 primers. With 4 primers, a LAMP product was detected after 60 min at 65°C (Fig. 2) while with 6 primers the amplification product was detected as early as 30 min (Fig. 3). KHV DNA extracted either by kit or by boiling gave rise to a typical ladder pattern: many bands of different size up to the loading well as shown in Figures 2, 3 and 5. After addition of 1 μl of diluted SYBR Green I to the reaction tube, positive reactions (amplified products) turned green, whereas all negative controls remained orange, the starting colour of SYBR Green (Fig. 4). The optimal primer concentration is stated in Methods.

### Specificity of the KHV LAMP primers and assay

**Figure 6:**
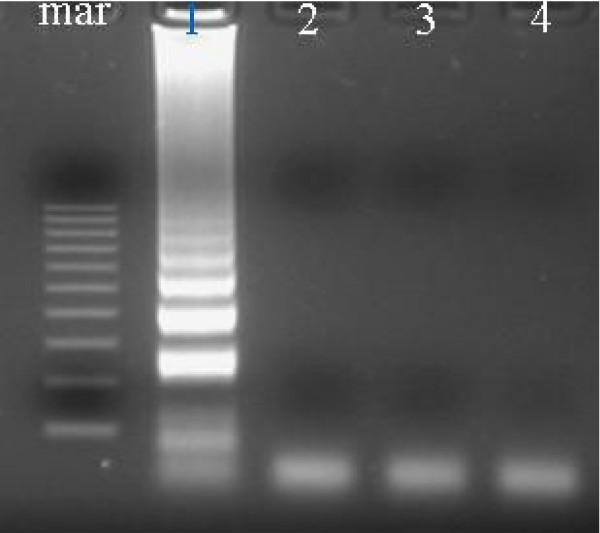
Agarose gel illustrating the specificity of the designed primers to KHV DNA. The reaction was carried out at 65°C using the 6 primer set for 1 hr. Lanes: 1 = KHV DNA; 2 = *Herpesvirus cyprini *(CHV) DNA showing no amplification; 3 = channel catfish virus (CCV) showing no amplification; 4 = uninfected koi tissue; mar = 100 bp DNA molecular weight marker.

Reaction products were detected only when KHV DNA was present, giving rise to a typical ladder-like pattern. There were no amplification products detected with *Herpesvirus cyprini *(CHV), channel catfish virus (CCV) or koi fish genomic DNA (Fig. 6).

### Sensitivity of the LAMP reaction in detection of KHV

**Figure 7:**
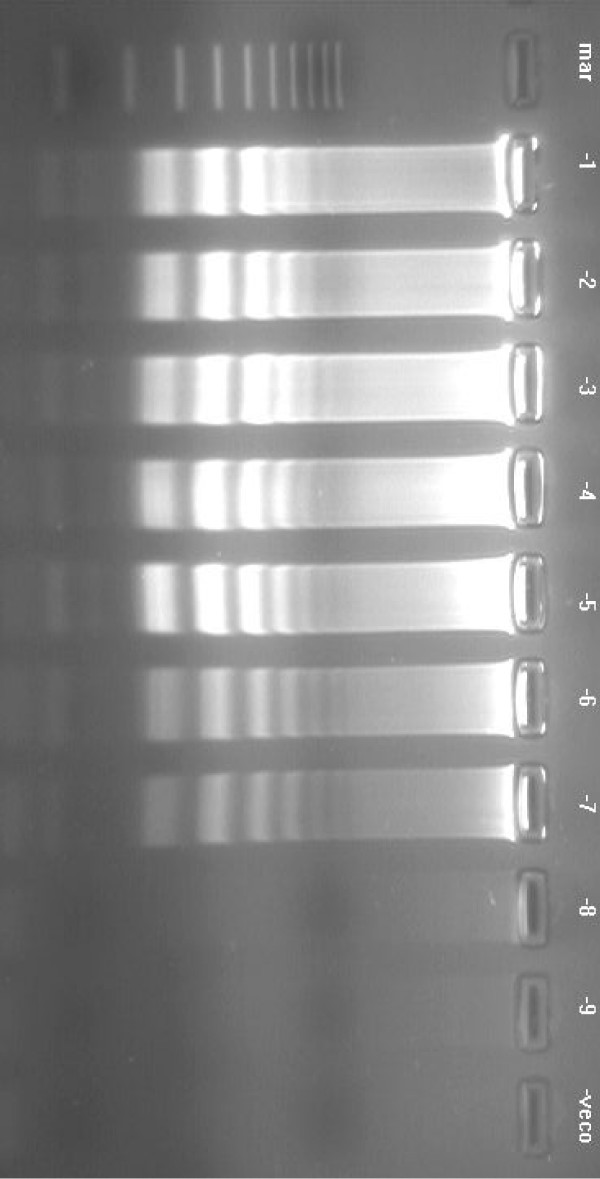
Agarose gel illustrating the sensitivity of the LAMP assay using 10-fold serial dilutions of purified KHV viral DNA. The amplification shows a ladder-like pattern, and detected purified KHV viral DNA down to a dilution of 10^7^. Lanes: -1 = dilution of 10^-1^; -2 = 10^-2 ^and so on; mar = 100 bp DNA molecular weight standard. -veco = negative control.

**Figure 8:**
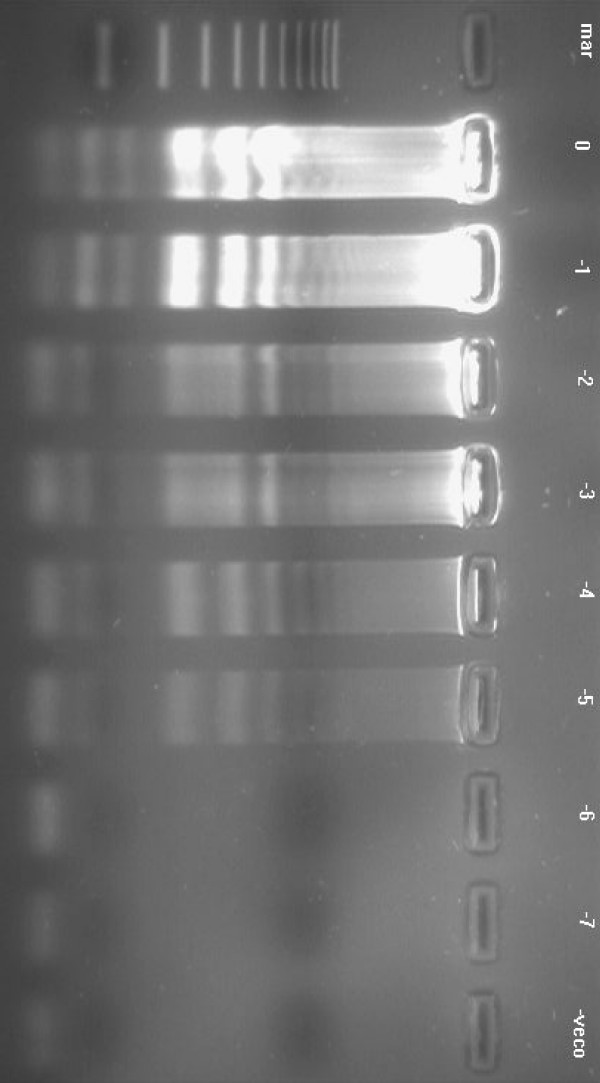
Agarose gel demonstrating the sensitivity of the LAMP assay using 10-fold serial dilutions of KHV DNA extracted from a clinical sample. The amplification shows a ladder-like pattern, and detected KHV DNA in a clinical sample at a dilution of 10^-5^. Lanes: 0 = undiluted KHV DNA; -1 = dilution of 10^-1^; -2 = 10^-2 ^and so on; mar = 100 bp DNA molecular weight standard. -veco = negative control.

**Figure 9:**
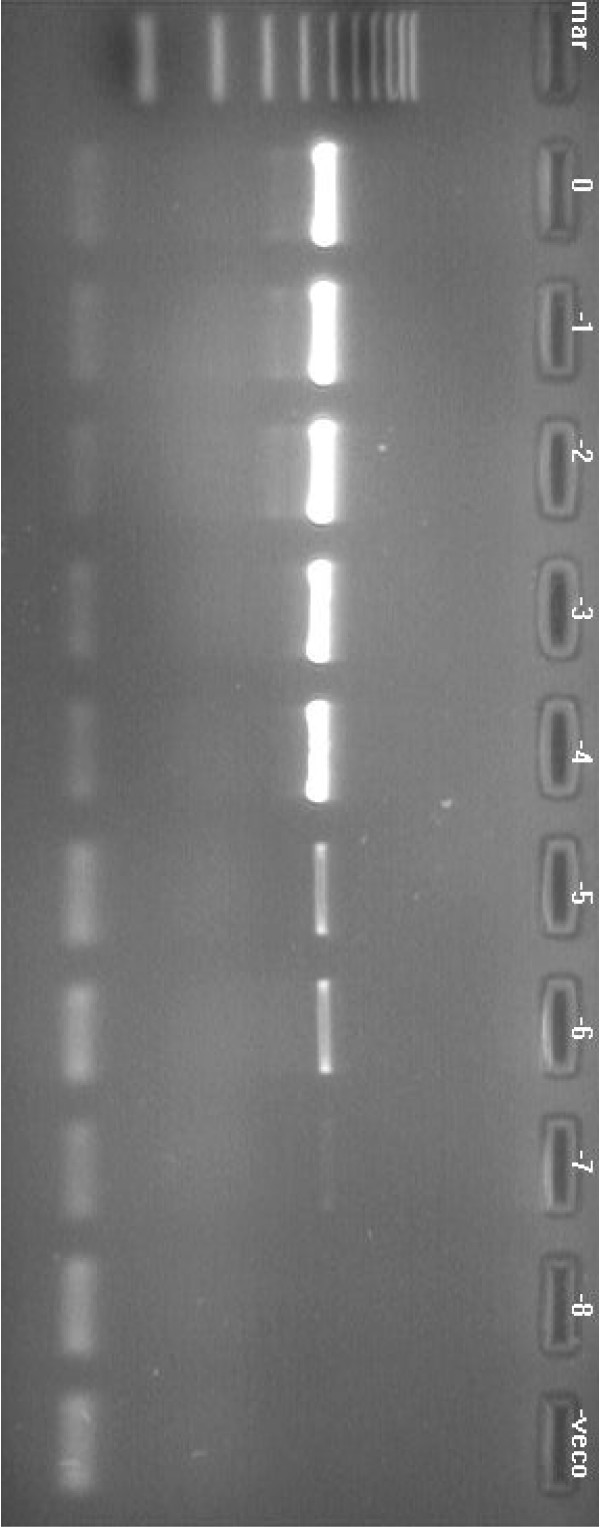
Agarose gel illustrating the sensitivity of the PCR assay using 10-fold serial dilutions of the purified KHV viral DNA. The PCR shows a 484 bp amplification product, and detected purified KHV viral DNA down to a dilution of 10^7^. Lanes: 0 = undiluted KHV DNA; -1 = dilution of 10^-1^; -2 = 10^-2 ^and so on; mar = 100 bp DNA molecular weight standard; -veco = negative control without target DNA.

**Figure 10:**
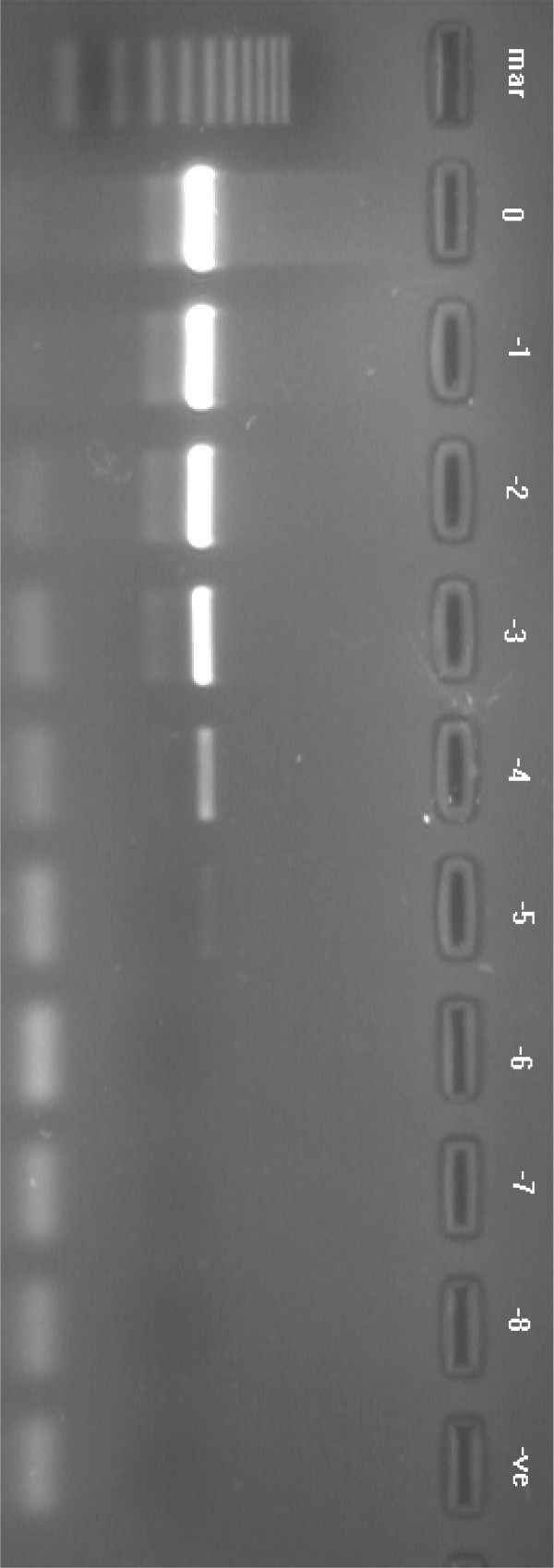
Agarose gel showing the sensitivity of the PCR assay using 10-fold serial dilutions of the KHV DNA extracted from a clinical sample. The PCR reveals a 484 bp amplification product, and detected KHV in a clinical sample at a dilution of 10^-5^. Lanes: 0 = undiluted KHV DNA; -1 = dilution of 10^-1^; -2 = 10^-2 ^and so on; mar = 100 bp DNA molecular weight standard; -ve = negative control without target DNA.

The reaction was tested using 10-fold serial dilutions of KHV DNA from both purified viral DNA and from DNA extracted from positive clinical samples, and compared against results from the commonly used PCR assay. The detection limit of both the LAMP and PCR assays using purified KHV viral DNA was 10^-7 ^(Fig. 7, 9). The detection limit of both assays was 10^-5 ^for clinical samples (Fig. 8, 10). These were the limits for the KHV LAMP reaction under optimal conditions: using 6 primers at 65°C for 60 min. If the reaction was run for 30 min, the detection limit of the LAMP assay was 10^-3 ^for the purified KHV viral DNA and 10^-1 ^for clinical samples. Increasing the primer concentrations did not affect these detection limits (data not shown).

### Applicability of the KHV LAMP reaction

50 clinical cases with suspected KHV infections were submitted to our laboratory and were investigated both with the LAMP assay and standard PCR. 37 out of 50 tested positive with both the PCR and LAMP; 13 were negative. No sample that was negative with the LAMP assay tested positive with the PCR, and vice versa.

## Discussion

The most extensively used diagnostic methods for KHV are cell culture and PCR. These techniques, however, require a relatively long time to produce results or are not practical for commercial producers, retailers, and regulators because of the equipment and expertise needed to conduct the assays. Moreover, the *Taq *DNA polymerase used in the PCR assay is easily inactivated by tissue- and blood-derived inhibitors such as myoglobin, hem-blood protein complex and immunoglobulin G [25-28]. Loop-mediated isothermal amplification (LAMP) is a novel method that facilitates rapid nucleic acid amplification using only simple equipment [13]. In the first step of the LAMP reaction, *Bst *polymerase synthesises new DNA between the F3 and B3 primers; this is the same reaction as standard PCR and requires homology between the primers and the template DNA. In the next step, the newly synthesised strands are recognised by the inner primers FIP and BIP to start loop mediated autocycling amplification [29] to produce stem-loop DNA structures with several inverted repeats of the target and cauliflower-like structures with multiple loops [22]. Amplification is specific and rapid when template which includes sequences that the loop primers recognise is present [20]. To accelerate the LAMP reaction 6 primers were used instead of 4. The two additional primers hybridised to the stem-loops (except for those loops that had been hybridized by the inner primers) [20].

KHV DNA extraction was performed by boiling fish tissues in a buffer solution; a simple and rapid technique [31-33] AL buffer was used to inactivate DNase and to elute DNA from tissues. Immediately after boiling, both undiluted and diluted DNA samples were trialled as templates for the LAMP reaction. No amplification products were detected for the undiluted DNA; addition of 800 μl TE buffer was necessary to dilute reaction inhibitors which were present in the boiled solution [30]. The LAMP assay was sensitive enough to detect KHV DNA at this (1:4) dilution. A specific type of DNA polymerase was required for the LAMP reaction, *Bst *DNA polymerase, which has two distinct activities: linear target isothermal multimerisation and amplification, and cascade rolling-circle amplification [34]. The mechanism of loop mediated isothermal amplification is similar to cascade rolling circle amplification. Occasionally, a different LAMP amplification pattern appeared as a result of linear target isothermal multimerisation and amplification, as LAMP primers and target DNA seem to randomly multimerize [29]. Betaine was used in the LAMP reaction mixture to reduce base stacking [35-37] and to increase not only the overall rate of reaction but also target selectivity by significantly reducing amplification of irrelevant sequences [13]. Use of SYBR Green I for visual inspection of LAMP amplification products was a simple and superior technique, with no gel electrophoresis and staining with ethidium bromide required. Only 1 μl of diluted SYBR Green I added to the reaction mixture was enough to see a result: if the reaction mix turned from orange to green it was judged as positive. This visualisation technique is effective due to the high specificity and amplification efficiency of LAMP [22].

The detection limit of the KHV LAMP reaction was determined through amplification of 10-fold serial dilutions of both purified KHV viral DNA and DNA from positive clinical samples (containing both fish and KHV DNA). The LAMP reaction was performed at 65°C for 30 and 60 min, and compared with the results of the standard PCR assay. There was no difference between the detection limit of the LAMP reaction and the PCR reaction at 60 min: both were positive at 10^-7 ^dilution of purified virus DNA, and at 10^-5 ^from the clinical samples. However, at 30 min the LAMP detected down to only 10^-3 ^dilution of viral DNA and 10^-1 ^dilution DNA from clinical samples. Hence the optimal LAMP conditions were determined to be 65°C for 60 min to detect KHV virus down to a concentration of 0.1 pg. Although the LAMP reaction had equivalent sensitivity to the PCR test, it is considered superior because it is a simpler technique which can be carried out in most situations where a rapid diagnostic method is required: under field conditions, in private clinics, and at quarantine inspection stations. A water bath is the only equipment needed, and is used for both the DNA extraction and nucleic acid amplification.

Although the application of LAMP for the detection of KHV has been reported previously [38], these authors use only 4 primers which target the KHV tk gene. In the current study, 6 primers which recognise 8 distinct regions on the KHV DNA were used, thereby enhancing the specificity of the reaction and eliminating false positive results [20]. Also, DNA extraction by boiling prior to the LAMP test and visualisation of reaction products using SYBR Green I DNA stain were employed to reduce the time needed to perform the KHV test and to simplify the procedure.

In conclusion, the KHV LAMP reaction is a highly sensitive, rapid, and reliable method that can be used under field condition for diagnosis of the KHV infection.

## Methods

### DNA oligonucleotides

**Figure 1:**
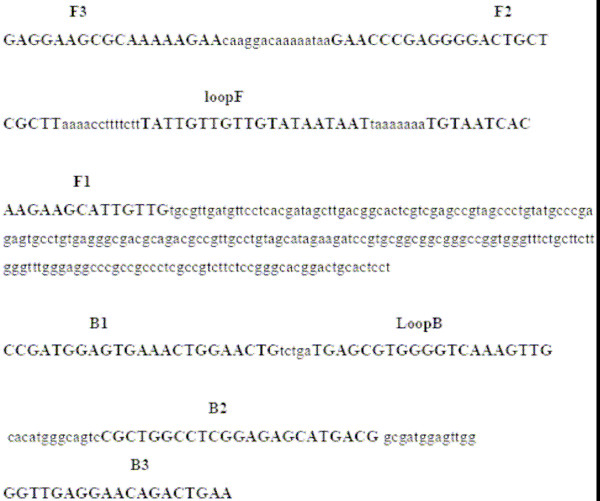
Nucleotide sequence of the KHV amplicon (GenBank accession number AF411803) used for construction of the inner and outer primers. The primer sequences are indicated in bold letters. Inner primers FIP and BIP comprise sequences within the amplicon; FIP is the complementary sequence of F1 and F2, BIP is B1 plus the complementary sequence of B2.

Six primers were designed from a KHV amplicon (Genbank Accession number AF411803), which recognise eight distinct regions of the target DNA. Forward inner primer (FIP) comprised the antisense sequence of F1 (23nt), a TTTT linker and a sense sequence of F2 (23nt): 5'- CAACAATGCTTCTTGTGATTACA-TTTT-GAACCCG AGGGGACTGCTCGCTT-3'. Backward inner primer (BIP) consisted of the sense sequence of B1 (23nt), a TTTT linker and the antisense sequence of B2 (23nt): 5'- CC GATGGAGTGAAACTGGAACTG-TTTT-CGTCATGCTCTCCGAGGCCAGCG-3'. The outer primers were F3 (19nt): 5'- GAGGAAGCGCAAAAAGAAC-3', and B3 (19nt): 5'- TTCAGTCTGTTCCTCAACC-3'. The loop primers were, loop F (20nt): 5'-ATTATTATAC AACAACAATA-3'; and loop B (20nt): 5'-TGAGCGTGGGGTCAAAGTT G-3'. (Fig. 1). Primers used in the PCR assay were constructed according to Gilad et al. (2002). Forward primer- KHV9/5F: 5'- GACGACGCCGGAGACCTTGTG-3', and reverse primer- KHV9/5R: 5'- CACAAGTTCAGTCTGTTCCTCAAC-3'. This primer set amplified a 484 bp segment of the KHV template.

### DNA extraction

Gills, kidney, spleen, and brain were sampled from fish sent to our laboratory with suspected KHV infections. DNA extraction was performed using both a commercial kit and a tissue boiling method. For the QIAamp DNA mini kit (QIAGEN GmbH, Hilden Germany), one gram of each organ was ground thoroughly in liquid nitrogen using a mortar and pestle. 20 mg of tissue powder was placed in a 2 ml microfuge tube, 180 μl of lysis buffer and 20 μl of proteinase K were added, then incubated at 56°C in a water bath until the tissues were completely lysed (1–3 h). DNA extraction was then completed according to the manufacturer's instructions, with final elution of DNA in 100 μl elution buffer, and storage at -20°C.

The second method of DNA extraction was by boiling: 20 mg of each tissue were placed in 2 ml microfuge tubes with 200 μl AL buffer (QIAGEN GmbH, Hilden, Germany), and placed in boiling water for 15 min. 800 μl of Tris- EDTA buffer (TE: 10 mM Tris-HCl, 0.1 mM EDTA, pH 8.0) was then added to the tube, mixed well, and centrifuged at 14,000 rpm for 3 min. The supernatant contained the DNA was used immediately in the KHV assays.

### LAMP reaction

The 25 μl reaction mixture comprised: 20 mM Tris-HCl (pH 8.8), 10 mM KCl, 6 mM MgSO_4_, 10 mM (NH_4_)_2_SO_4_, 0.1% Triton X-100, 1.6 M betaine, deoxynucleotide triphosphates 2.8 mM each, 1.6 μM each FIP and BIP, 0.8 μM each loop-F and loop-B, 0.2 μM each F3 and B3 primers, 8 U *Bst *DNA polymerase (New England BioLabs, GmbH, Frankfurt, Germany), 2 μl template DNA, distilled water to 25 μl. As a negative control, template DNA was omitted from the reaction. The mix was incubated at 65°C for 60 min and then heated at 80°C for 2 min to terminate the reaction.

### Analysis of LAMP products

1 μl of 1:10 diluted SYBR Green I Nucleic acid gel stain, 10,000× concentration in DEMSO (Cambrex Bio Science, Rockland, Inc, ME USA) was added directly to the reaction tube and any colour change observed. The solution turned green if LAMP reaction products were present, otherwise it remained orange. Reaction products were also analysed by gel electrophoresis: 5 μl aliquots were analysed on a 2% agarose gel and subsequently stained with ethidium bromide; a DNA molecular weight marker, 100 bp DNA Ladder, (Cambrex Bio Science, Inc, Rockland, ME USA) was used to determine the size of the products.

### PCR assay

Amplification was performed according to Gilad et al. (2002) in a standard reaction volume of 50 μl comprising 3 μl template DNA and 47 μl 1.1× ReaddyMix PCR Master mix: 75 mM Tris-HCl (pH 8.8), 20 mM (NH_4_)_2_SO_4_, 1.5 mM MgCl_2_, 0.01% Tween20, 0.2 mM each of dATP, dCTP, dGTP, dTTP, 1.25 U *Taq *DNA polymerase and red dye for electrophoresis (ABgene, Hamburg, Germany) and forward and reverse primers (20 pmol each). The reaction mixture was subjected to 39 amplification cycles under the following conditions: denaturation at 94°C for 1 min, annealing at 68°C for 1 min, extension at 72°C for 30s. The amplification cycles were preceded by a denaturation step at 94°C for 5 min and followed by an extended elongation step at 72°C for 7 min.

### Detection of PCR products

products were analysed by electrophoresis on 1.5% agarose gels stained with ethidium bromide. 100 bp DNA Ladder (Cambrex Bio Science, Inc, Rockland, ME USA) was used to determine the size of the PCR products.

### Optimisation of KHV LAMP reaction conditions

varying concentrations of the FIP, BIP, F3, B3, loop-F, loop-B primers were trialled, as well as use of only 4 primers (excluding loop-F and loop-B). Time of reaction was varied in 5 minute increments from 10–60 min to determine detection time of KHV genomic DNA.

### Specificity of the KHV LAMP assay

The reaction was tested using DNA from *Herpesvirus cyprini *(CHV), channel catfish virus (CCV) and koi genomic DNA.

### Sensitivity of the KHV LAMP reaction

The detection limits of the KHV LAMP assay were evaluated using 10-fold serial dilutions of purified KHV DNA and DNA extracted from positive clinical samples. The reaction was performed at 65°C for both 30 and 60 min, and compared with the PCR assay results.

### Applicability of the KHV LAMP reaction

After the initial validation studies, the KHV LAMP reaction was used to test 50 suspected clinical cases submitted to our laboratory and the results compared with the PCR assay results of those 50 cases.

## Author's contributions

ME conceived and supervised the study and drafted the manuscript. HS carried out all the experimental work and data acquisition.

## References

[B1] Hedrick RP, Gilad O, Yun S, Spangenberg JV, Marty GD, Nordhausen RW, Kebus MJ, Bercovier H, Eldar A (2000). A herpesvirus associated with mass mortality of juvenile and adult koi, a strain of common carp. J Aquat Anim Health.

[B2] Ronen A, Perelberg A, Abramowitz J, Hutoran M, Tinman S, Bejerano I, Steinitz M, kotler M (2003). Efficient vaccine against the virus causing a lethal disease in cultured *Cyprinus carpio*. Vaccine.

[B3] Waltzek TB, Kelley GO, Yun SC, McDowell TS, Hedrick RP (2004). Relationships of Koi Herpesvirus (KHV) to Herpes-like Viruses of Fish and Amphibians. Proceedings, 35th Annual Conference International Association for Aquatic Animal Medicine, Galveston, TX.

[B4] Hedrick RP (1996). Movement of pathogens with the international trade of live fish; Problems and solutions. Rev Sci Tech.

[B5] Gilad O, Yun S, Adkison M, Way K, Willits N, Bercovier H, Hedrick RP (2003). Molecular comparison of isolates of an emerging fish pathogen, koi Herpesvirus, and the effect of water temperature on mortality of experimentally infected koi. J Gen Virol.

[B6] Bretzinger A, Fischer-Scherl T, Oumouna M, Hoffmann R, Truyen U (1999). Mass mortalities in koi, *Cyprinus carpio*, associated with gill and skin disease. Bull Eur Ass Fish Pathol.

[B7] Oh MJ, Jung SJ, Choi TJ, Kim HR, Rajendran KV, Kim YJ, Park MA, Chun SK (2001). A viral disease occurring in cultured carp *Cyprinus carpio *in Korea. Fish pathol.

[B8] Choi D, Sohn S, Bang J, Do J, Park M (2004). Ultrastructural identification of a herpes-like virus infection in common carp *Cyprinus carpio *in Korea. Dis Aquat Org.

[B9] Rukyani A (2002). Koi Herpesvirus infection in Indonesia. ProMed Jun 30.

[B10] Miyazaki T, Okamoto H, Kageyama T, Kobayashi T (2003). Viremia associated ana-aki-byo, a new viral disease in colour carp *Cyprinus carpio *in Japan. Dis Aquat Org.

[B11] Hartman KH, Yanong RP, Petty BD, Francis-Floyd R, Riggs AC (2004). Koi Herpes Virus (KHV) Disease. Fact sheet VM-149, Extension service, Institute of Food and Agricultural Sciences, University of Florida.

[B12] Gray WL, Mullis L, LaPatra SE, Groff JM, Goodwin A (2002). Detection of Koi herpesvirus DNA in tissues of infected fish. J Fish Dis.

[B13] Notomi T, Okayama H, Masubuchi H, Yonekawa T, Watanabe K, Amino N, Hase T (2000). Loop-mediated isothermal amplification of DNA. Nuc Acids Res.

[B14] Saiki RK, Scharf S, Faloona F, Mullis KB, Horn GT, Erlich HA, Arnheim N (1985). Enzymatic amplification of beta-globin genomic sequences and restriction site analysis for diagnosis of sickle cell anaemia. Science.

[B15] Saiki RK, Gelfand DH, Stoffel S, Scharf S, Higuchi R, Horn GT, Mullis KB, Erlich HA (1988). Primer-directed enzymatic amplification of DNA with a thermostable DNA polymerase. Science.

[B16] Gilad O, Yun S, Andree KB, Adkison MA, Zlotkin A, Bercovier H, Eldar A, Hedrick RP (2002). Initial characteristics of koi Herpesvirus and development of a polymerase chain reaction assay to detect the virus in koi, *Cyprinus carpio koi*. Dis Aquat Org.

[B17] Gilad O, Yun S, Zagmutt-Vergara FJ, Leutenegger CM, Bercovier H, Hedrick RP (2004). Concentrations of a Koi herpesvirus (KHV) in tissues of experimentally-infected *Cyprinus carpio koi *as assessed by real-time TaqMan PCR. Dis Aquat Org.

[B18] Nagamine K, Watanabe K, Ohtsuka K, Hase H, Notomi T (2001). Loop-mediated isothermal amplification reaction using a nondenatured template. Clin Chem.

[B19] Mori Y, Nagamine K, Tomita N, Notomi T (2001). Detection of loop-mediated isothermal amplification reaction by turbidity derived from magnesium pyrophosphate formation. Biochem Biophys Res Commun.

[B20] Nagamine K, Hase T, Notomi T (2002). Accelerated reaction by loop-mediated isothermal amplification using loop primers. Mol Cell Probes.

[B21] Ihira M, Yoshikawa T, Enomoto Y, Akimoto S, Ohashi M, Suga S, Nishimura N, Ozaki T, Nishiyama N, Ozaki T, Nishiyama Y, Notomi T, Ohta Y, Asano Y (2004). Rapid diagnosis of Human Herpesvirus 6 infection by a novel DNA amplification method, loop-mediated isothermal amplification. J Clin Microbiol.

[B22] Iwamoto T, Sonobe T, Hayashi K (2003). Loop-mediated isothermal amplification for direct detection of *Mycobacterium tuberculosis *complex, *M. avium*, and *M. intracellulare *in sputum samples. J Clin Microbiol.

[B23] Waltzek TB, Hedrick RB Koi Herpesvirus update. California Veterinarian.

[B24] Way K, LeDeuff RM, Stone DM, Denham KL, St-Hilaire S (2004). Koi Herpesvirus diagnosis and research at CEFAS Weymouth laboratory 2000–2003. International workshop on koi Herpesvirus.

[B25] Belec L, Authier J, Eliezer-Vanerot M, Piedouillet C, Mohamed A, Gherardi R (1998). Myoglobin as a polymerase chain reaction (PCR) inhibitor: a limitation for PCR from skeletal muscle tissue avoided by the use of *Thermus thermophilus *polymerase. Muscle and Nerve.

[B26] Akane A, Matsubara K, Nakamura H, Takahashi S, Kimura K (1994). Identification of the heme compound copurified with deoxyribonucleic acid (DNA) from bloodstains, a major inhibitor of polymerase chain reaction (PCR) amplification. J Forensic Sci.

[B27] Al-Soud W, Önsson LJ, Radström P (2000). Identification and characterization of immunoglobulin G in blood as a major inhibitor of diagnostic PCR. J Clin Microbiol.

[B28] Johnson S, Martin D, Cammarata C, Morse S (1995). Alterations in sample preparation increase sensitivity of PCR assay for diagnosis of chancroid. J Clin Microbiol.

[B29] Kuboki N, Inoue N, Sakurai T, Di Cello F, Grab DJ, Suzuki H, Sugimoto C, Igarashi I (2003). Loop-mediated isothermal amplification for detection of African trypanosomes. J Clin Microbiol.

[B30] Ikeda N, Bautista N, Yamada T, Kamijima O, Ishii T (2001). Ultra-simple DNA extraction method for marker-assisted selection using microsatellite markers in rice. Plant Mol Biol Rep.

[B31] Valsecchi E (1998). Tissue boiling: a short-cut in DNA extraction for large-scale population screening. Mol Ecol.

[B32] Sepp R, Szabo I, Uda H, Sakamoto H (1994). Rapid techniques for DNA extraction from routinely processed archival tissue for use in PCR. J Clin Pathol.

[B33] Afghani B, Stutman HR (1996). Polymerase chain reaction for diagnosis of *M. tuberculosis*: comparison of simple boiling and conventional methods for DNA extraction. Biochem Mol Med.

[B34] Hafner GJ, Yang IC, Wolter LC, Stafford MR, Giffard PM (2001). Isothermal amplification and multimerization of DNA by *Bst *DNA polymerase. BioTechniques.

[B35] Rees WA, Yager TD, Korte J, Von Hippel PH (1993). Betaine can eliminate the base pair composition dependence of DNA melting. Biochemistry.

[B36] Baskaran N, Kandpal RP, Bhargava AK, Glynn MW, Bale A, Weissman SM (1996). Uniform amplification of a mixture of deoxyribonucleic acids with varying GC content. Genome Res.

[B37] Rajendrakumar CS, Suryanarayana T, Reddy AR (1997). DNA helix destabilization by proline and betaine: possible role in the salinity tolerance process. FEBS Letters.

[B38] Gunimaladevi I, Kono T, Venugopal MN, Sakai M (2004). Detection of koi Herpesvirus in common carp, *Cyprinus carpio L*., by loop-mediated isothermal amplification. J Fish Dis.

